# Closed-Cell Rigid Polyimide Foams for High-Temperature Applications: The Effect of Structure on Combined Properties

**DOI:** 10.3390/polym13244434

**Published:** 2021-12-17

**Authors:** Yawei Shi, Aijun Hu, Zhiyuan Wang, Kedi Li, Shiyong Yang

**Affiliations:** 1 Key Laboratory of Science and Technology on High-Tech Polymer Materials, Institute of Chemistry, Chinese Academy of Sciences, Beijing 100190, China; shiywei@iccas.ac.cn (Y.S.); zhiyuan@iccas.ac.cn (Z.W.); 2 School of Chemical Sciences, University of Chinese Academy of Sciences, Beijing 100190, China; 3 Cashem Advanced Materials Hi-Tech Co., Ltd., Shaoxing 312369, China; ked.li@cashem.cn

**Keywords:** polyimide foam, rigid closed-cell foam, high temperature, benzimidazole

## Abstract

Closed-cell rigid polyimide foams with excellent thermal stability and combined properties were prepared by thermal foaming of a reactive end-capped polyimide precursor powder in a closed mold. The precursor powder was obtained by thermal treatment of a polyester-amine salt (PEAS) solution derived from the reaction of the diethyl ester of 2,3,3′,4′-biphenyl tetracarboxylic dianhydride (α-BPDE) with an aromatic diamine mixture of p-phenylenediamine (PDA) and 2-(4-aminophenyl)-5-aminobenzimidazole (BIA) in the presence of an end-capping agent (mono-ethyl ester of nadic acid anhydride, NE) in an aliphatic alcohol. The effect of polymer mainchain structures on the foaming processability and combined properties of the closed-cell rigid polyimide foams were systematically investigated. The polyimide foams (100–300 kg/m^3^) with closed-cell rates of 91–95% show an outstanding thermal stability with an initial thermal decomposition temperature of ≥490 °C and a glass transition temperature of 395 °C. Polyimide foams with density of 250 kg/m^3^ exhibited compression creep deformation as low as 1.6% after thermal aging at 320 °C/0.4 MPa for 2 h.

## 1. Introduction

Polyimide (PI) foams [1,2,3,4], due to the presence of the imide groups in the polymer mainchain, display excellent thermal stability, thermal insulating properties, non-flammability, radiation resistance, etc. and have found widespread application in aerospace, aviation, orbiting vehicles, and transportation [5,6,7]. There are two types of PI foams based on the cell morphology: an open-cell version and a closed-cell one [8,9]. Generally, most open-cell PI foams are soft. Closed-cell PI foams may have different closed-cell rates of less than 100%. The commercial SOLIMIDE^®^ foams (BOYD Corporation) are a series of open-cell soft PI foams with density of 5 to 64 kg/m^3^, which can be maintained in long-term service at temperatures up to 300 °C [9,10]. Due to the open-cell structure and low strength, open-cell soft PI foams are usually used as heat insulation and sound absorbing materials. With the development of the aerospace industry, lightweight and high-strength foam materials have been required for applications such as cryogenic insulation and energy absorption. For example, the production of re-usable launch vehicles (RLVs) requires high performance structural insulation foam materials which may be subject to service temperatures ranging from −250 °C to 250 °C [11]. The construction of high speed aerospace vehicles requires a core-splice material with a density of 500 kg/m^3^ and a potting material with a density of 560 kg/m^3^, respectively [12]. Hence, rigid PI foams with excellent thermal stability and combined mechanical properties are required for application in the aerospace field.

DuPont started research on PI foams as early as the 1960s and later launched commercial rigid PI foams under the trade name Vespel^®^ [13,14]. Vespel^®^ PI foams possess an excellent compressive strength of 2.0 MPa with a density (*ρ*) of 300 kg/m^3^. The maximum service temperature is about 300 °C. The Langley Research Center at NASA has prepared a series of closed-cell PI foams with closed-cell rates of <80% by foaming a salt-like precursor [9,15]. The salt-like precursor was synthesized by mixing the dimethyl ester of a dianhydride and a diamine in methanol. The compressive strength of the foam is in the range of 0.098–0.84 MPa, and the glass transition temperature (Tg) is ≤321 °C.

To improve the performance of PI foams, the incorporation of inorganic fillers such as silica, graphene, and carbon fibers [8,16,17,18,19,20,21] into the foam matrix has been utilized. PI/organoclay nanocomposite foams have been prepared through thermal foaming of a polyester amine salt (PEAS)/organoclay precursor [22]. Hydrogen bonding between the organoclay and the polymer matrix provided enhancements of both the Tg and decomposition temperature. However, property improvements were limited by the required dosage of the fillers, as the presence of fillers usually have negative effect on cell morphology [16].

The introduction of a cross-linked structure in the polyimide matrix can restrict the mobility of polymer chains, resulting in improvements in thermal stability and mechanical properties [23,24,25,26,27,28,29]. A series of crosslinked PI foams were prepared by copolymerization of a trifunctional diamine monomer 2,4,6-triaminopyrimidine (TAP) with BTDA-ODA to form a PI foam [23,24]. Both the Tg and tensile strength of the PI foam were increased. The PI foams (PIF-15) with 15% content of TAP displayed a Tg of 286.7 °C, compared with 272.6 °C for PIF-0 containing no TAP. A thermosetting/thermoplastic PI foam was prepared using in situ simultaneous orthogonal polymerization [25,30]. The precursor of linear PI and the monomer of cross-linked bismaleimide (BMI) were blended and foamed at high temperature. The Tg values for the PI foams increased with increasing BMI content, from 255.85 °C for neat PI foam to 285.12 °C for PIF-30 with a BMI content of 30 wt.%.

An approach for the production of closed-cell rigid PI foams by thermal foaming of nadimide end-capped polyimide oligomers (NPOs) has been reported. The NPOs were obtained by thermally treating an in situ polymerization of a monomeric reactant (PMR)-type polyester-amine salt (PEAS) solution [26,27]. The PEAS was synthesized from the reaction of the diethyl ester of aromatic dianhydride (α-BPDE) and aromatic diamines using mono-ethyl ester of cis-5-norbornene-endo-2,3-dicarboxylic anhydride (NE) as the reactive end-capping agent in ethyl alcohol. A crosslinking structure was formed through a reverse Diels–Alder reaction of the end-capped groups during the foaming process. The resulting rigid PI foams contained a uniform cellular structure with a closed-cell rate as high as 89%, along with a high thermal stability and good mechanical properties.

Heteroaromatic diamine monomers containing benzimidazole or the benzoxazole structure have also been employed as co-monomers to improve the closed-cell rigid PI combined properties. The incorporation of benzimidazole or the benzoxazole structure can increase the rigidity of polymer molecular segments and promote the formation of hydrogen bonds between polymer chains [28,31]. A series of PI foams containing the comonomer 2-(4-aminophenyl)-5-aminobenzoxazole (DAPBO) have been prepared [32]. The rigid PI foam with 60% DAPBO content displayed a Tg value of 368 °C, which was much higher than that of the pristine PI foam (273 °C). The compressive strength was also increased to 1.03 MPa from 0.3 MPa. Obviously, the introduction of a heterocyclic structure or crosslinked structure can improve the combined properties of the PI foams.

A heteroaromatic diamine, 2-(4-aminophenyl)-5-aminobenzimidazole (BIA), was employed as a comonomer to improve the combined properties of closed-cell PI foams possessing a crosslinked structure. The PI foams were produced by the thermal foaming of the nadimide end-capped polyimide oligomers (NPOs), which were prepared by the copolymerization of an aromatic dianhydride (α-BPDA) and an aromatic diamine mixture containing BIA and PDA using cis-5-norbornene-endo-2,3-dicarboxylic anhydride (NA) as the end-capping agent. The BIA content and the molecular weight (M_w_) of the PI precursors had a significant effect on the cell morphology and overall performance of the PI foams.

## 2. Materials and Methods

### 2.1. Materials

2,3,3′,4′-Biphenyl tetracarboxylic dianhydride (α-BPDA) was purchased from China-tech (Tianjin) Chemical Co., Ltd., (Tianjin, China) and dried at a reduced pressure at 160 °C for 8 h prior to use. *cis*-5-Norbornene-*endo*-2,3-dicarboxylic anhydride (NA) was purchased from Changzhou Sunlight Pharmaceutical Co., Ltd., (Changzhou, China) and dried at a reduced pressure at 80 °C for 8 h prior to use. p-Phenylenediamine (PDA) and 2-(4-aminophenyl)-5-aminobenzimidazole (BIA) were obtained from Changzhou Sunlight Pharmaceutical Co., Ltd., (Changzhou, China) and used as received. Ethyl alcohol was obtained from Beijing Chemical Reagents Co. (Beijing, China) and stored over 4 Å molecular sieves to remove the moisture.

### 2.2. Preparation of Nadimide End-Capped Polyimide Oligomers (NPOs)

α-BPDA (0.1 mol) (29.42 g) and NA (0.07 mol) (11.49 g) were combined in 63.58 g of ethanol (1.38 mol). The solution was stirred at solvent reflux for three hours to obtain a homogeneous solution. Then, 11.68 g of PDA (0.108 mol) and 6.06 g of BIA (0.027 mol) were added, and the solution was stirred at solvent reflux for another two hours to produce a homogeneous PEAS solution. After removing most of the solvent by rotating distillation, the viscous liquid was dried at a reduced pressure at 200 °C. The obtained solid resin was crushed into a fine powder and sieved, yielding a powder with a particle diameter of 50 to 200 μm. In this experiment, the oligomer powder (NPO-2) had a BIA/(BIA + PDA) molar ratio of 20% and a calculated molecular weight (calcd M_w_) of 1500 g/mol.

In addition, a series of NPO powders (NPO-0, NPO-1, and NPO-3) with a fixed calcd M_w_ of 1500 g/mol but different BIA/(BIA + PDA) molar ratios of 0%, 10%, and 30%, respectively, were prepared by adjusting the molar ratio of aromatic diamines. Moreover, a series of NPO powders with a fixed molar ratio of BIA/(BIA + PDA) (20%) but different calcd M_w_ were also prepared, including NPO-2-1000 (calcd M_w_ = 1000), NPO-2-1250 (calcd M_w_ = 1250), NPO-2-1500 (calcd M_w_ = 1500), NPO-2-1750 (calcd M_w_ = 1750), and NPO-2-2000 (calcd M_w_ = 2000), respectively.

### 2.3. Preparation of Closed-Cell Rigid PI Foams

The polyimide foams were prepared by thermal foaming of the NPO powder in a closed mold. The NPO powder was placed at the bottom of the mold, which was then heated in a hot press. The mold was heated stepwise to 350 °C and held for three hours with applied pressure. Then, the mold was cooled to room temperature and the rigid PI foam was removed and cut into the desired dimensions for testing.

The amount of NPO powder placed in the mold was determined according to the desired foam density and mold volume as well as the calculated amounts of organic volatiles evolved in the thermal foaming process. The foam density was determined by the amount of precursor and the size of the mold:

*ρ* = a × m/V (1)

where a is a constant factor determined by the mass loss during the foaming process and the volume shrinkage of the foam caused by the cooling process, m is the mass of the NPO precursor, and V represents the volume of the mold.

Hence, a series of rigid PI foams (PIF-0, PIF-1, PIF-2, and PIF-3) with a fixed calcd M_w_ of 1500 g/mol but different molar ratios of BIA/(BIA + PDA) of 0%, 10%, 20%, and 30% were prepared, respectively. Similarly, a series of rigid PI foams (PIF-2-1000, PIF-2-1250, PIF-2-1500, PIF-2-1700, and PIF-2-2000) with a fixed molar ratio of BIA/(BIA + PDA) of 20% but different calcd M_w_ were also prepared. Additionally, a series of rigid PI foams with a fixed BIA/(BIA + PDA) molar ratio of 20% and calcd M_w_ of 1500 g/mol but different densities (70, 150, 200, and 300 kg/m^3^) were prepared. Unless otherwise indicated, the density of the foam was 100 kg/m^3^.

### 2.4. Measurements

The chemical structures of the NPO precursors were characterized using a Bruker TENSOR27 Fourier transform infrared (FT-IR) spectrometer (Bruker, Karlsruhe, Germany). The measurements were performed in the range of 400–4000 cm^−1^ by averaging 32 scans. ^1^ H NMR spectra were obtained on a Bruker AVANCE 300 spectrometer (Bruker, Karlsruhe, Germany) using dimethyl sulfoxide (DMSO-d6) (J&K Co, Beijing, China) as a solvent. The morphology of the PI foam cellular structure was observed using a Hitachi S-4800 scanning electron microscope (SEM) (Hitachi, Tokyo, Japan). The closed-cell rate of the PI foams was measured by a Micromeritic AccuPyc II 1340 pycnometer (Micromeritics, Norcross, GA, USA) according to GB/T 10799-2008 using specimens with the dimensions 25 mm × 25 mm × 25 mm and under 2.9 psi pressure.

The rheological behavior measurements of the NPO precursors at different temperatures were performed on a TA AR2000 rheometer (TA Instruments, New Castle, DE, USA) with a heating rate of 4 °C/min. The specimen disks (25 mm in diameter and weighing 1 g) were prepared by compression molding of the NPO powder. The rheometer instrument was equipped with a 25 mm diameter parallel plate fixture. The upper plate was oscillated at a fixed strain of 0.1% and a constant angular frequency of 10 rad/s, while the lower plate was attached to a transducer that recorded the resultant torque and converted it to the complex viscosity.

Thermal gravimetric analysis (TGA) was performed on a TA Q50 thermal analysis system (TA Instruments, New Castle, DE, USA) with a heating rate of 20 °C/min under an air atmosphere with a gas flow rate of 40 mL/min. Dynamic mechanical analysis (DMA) was performed on a TA Q800 instrument (TA Instruments, New Castle, DE, USA) under an N_2_ atmosphere with a heating rate of 5 °C/min. A dual cantilever mode was employed using specimens with the dimensions 60.0 mm × 15.0 mm × 5.0 mm.

The mechanical properties of the PI foams were measured using an Instron 5567 universal testing machine (Instron, Norwood, MA, USA). The compression property measurement was performed according to GB T8813-2008 using specimens with a size of 25 mm × 25 mm × 25 mm at a compression rate of 2.5 mm/min. Dumbbell-shaped specimens were prepared to test the tensile properties of the PI foams with a tensile rate of 5 mm/min according to GB/T 9641-1988.

## 3. Results and Discussion

### 3.1. Characterization

**Figure 1:**
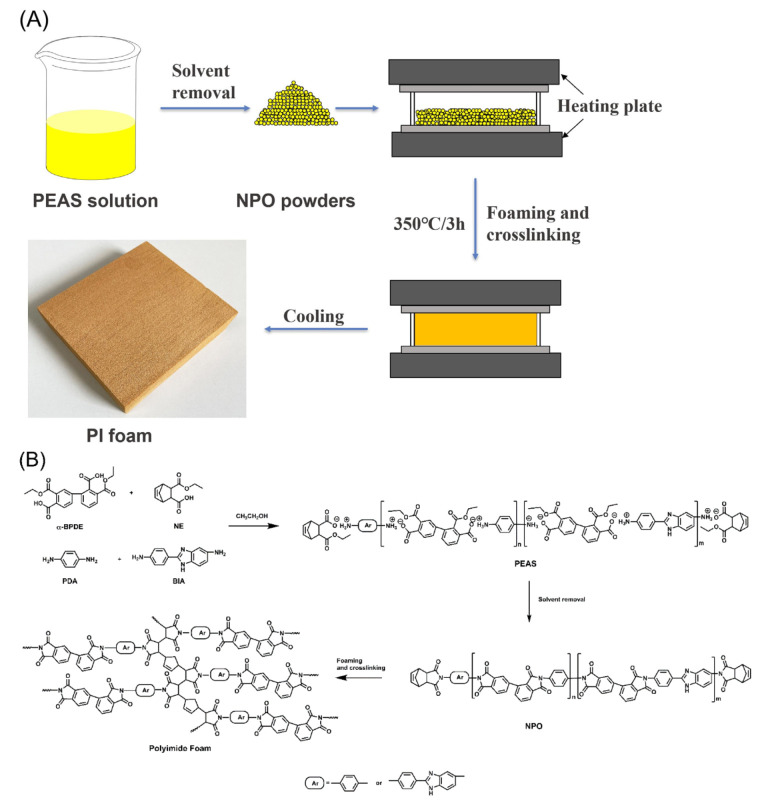
(**A**) Schematic diagram and (**B**) chemical reaction formula of procedure to fabricate the rigid PI foams. α-BPDE, diethyl ester of 2,3,3′,4′-biphenyl tetracarboxylic dianhydride; NE, monoethyl ester of *cis*-5-norbornene-endo-2,3-dicarboxylic anhydride. α-BPDE and NE were obtained by reflux esterification of corresponding monomers in ethanol. The foam dimensions were 220 mm × 220 mm × 35 mm.

The rigid PI foams were prepared by thermal foaming of the reactive end-capped NPO precursor powder at 350 °C for 3 h in a closed mold. As shown in Figure 1, the NPO powder was obtained by thermal treating of the polyester–amine salt (PEAS) solution derived from the reaction of the α-BPDA diethyl ester with PDA and BIA in the presence of an end-capping agent (monoethyl ester of nadic acid anhydride, NE) in aliphatic alcohol. The prepared foam showed a dense cell structure with a light-yellow color after the high-temperature foaming process.

**Figure 2:**
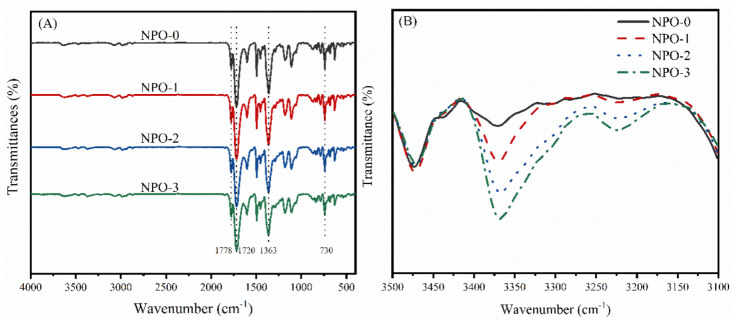
FT-IR spectra of NPOs with different BIA contents: (**A**) complete spectrum and (**B**) partial spectrum at 3100–3500 cm^−1^.

The FT-IR spectra of the NPOs with different BIA contents are shown in Figure 2. The NPOs showed imide ring characteristic absorption peaks at 1778 cm^−1^ (C=O asymmetric stretching vibration), 1720 cm^−1^ (C=O symmetric stretching vibration), 1363 cm^−1^ (C–N stretching vibration), and 730 cm^−1^ (imide ring bending vibration), indicating the formation of an imide structure (Figure 2A). The peaks at 3100–3450 cm^−1^ were attributed to the asymmetric and symmetric stretching vibration of N–H as shown in Figure 2B [33,34]. The peak intensity increased with increasing BIA content in the NPOs, indicating the presence of benzimidazole moieties in the polymer backbones.

**Figure 3:**
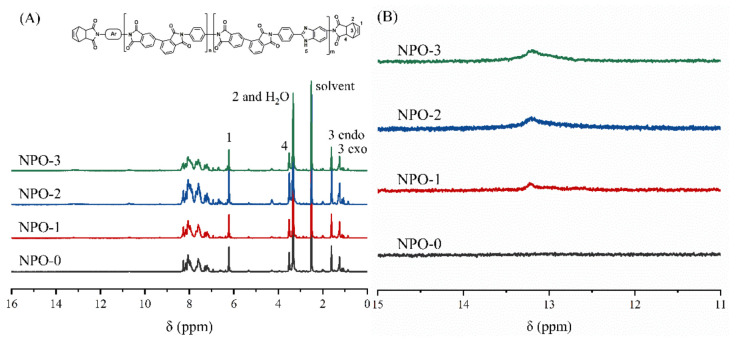
NMR spectra of NPOs with different BIA contents: (**A**) complete spectrum and (**B**) partial spectrum at 11–15 ppm.

The chemical structures of the NPOs were further confirmed by NMR spectra (Figure 3). The signals for H_1_ to H_4_ in the NA end-capping agent (Figure 3A) were detected in all NPO precursors. The signals at 13.26 ppm were attributed to H_5_ in the BIA moiety, and the intensity was increased with the increase in BIA content. These results confirm the existence of the NA end-capping groups and BIA moiety in the polymer backbones.

The molecular weights of NPO-2 with a fixed BIA content of 20% and different calcd M_w_ of 1000–2000 g/mol were measured by GPC (Table 1). The measured M_n_ values showed the same trend as the calcd M_w_, and the determined polydispersity ranged from 1.45 to 1.53.

### 3.2. Thermal Foaming Processability

**Figure 4:**
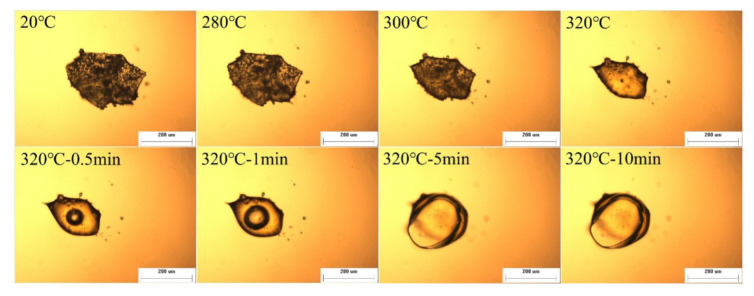
Foaming process of NPO-2 with increasing temperature.

The thermal foaming process of NPO-2 was monitored using a hot stage microscope with a heating rate of 10 °C/min. The temperature was increased to 320 °C, and the corresponding morphologies of the NPO powder at different temperatures are shown in Figure 4. The morphology of the NPO powder remained unchanged at temperatures below 280 °C, and some volume shrinkage was observed at 300 °C. At 320 °C, the NPO powder was completely melted and bubbles began forming. The foam bubbles gradually expanded from 0.5 to 10 min, implying that there was adequate strength maintaining the bubbles’ intactness. The strength of the melt could be attributed to the crosslinking reaction of NPO during the foaming process [27]. In the foaming process, two types of foaming agents are produced: the byproducts of the imidization reaction, such as H_2_O and CH_3_CH_2_OH, and the cyclopentadiene yielded by the thermal decomposition of the NA end-capping groups [35,36].

**Figure 5:**
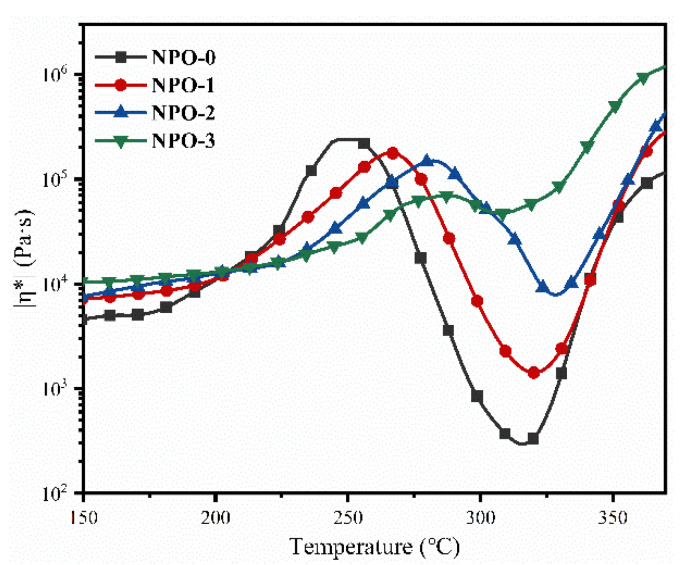
Rheological behaviors of melting for NPOs with different BIA contents.

Figure 5 compares the effect of BIA content on the NPO complex melt viscosities at 150–400 °C. The NPO powders began melting at temperatures higher than 250 °C; the melt viscosities decreased with the increase in temperature. After the minimum value, the melt viscosities were markedly increased due to the thermal crosslinking of the end-capping groups with a further increase in temperature, and the minimum melt viscosity was observed at 300–330 °C. Table 2 summarizes the minimum melt viscosity and the corresponding temperatures of the NPO powders with different BIA contents. Obviously, the minimum melt viscosity increased with the increase in BIA content in the polymer backbones. For instance, NPO-0 showed a minimum melt viscosity of 233 Pa·s at 317.3 °C, lower than that of NPO-1 (826 Pa·s at 324.1 °C), NPO-2 (5134 Pa·s at 328.9 °C), and NPO-3 (45,490 Pa·s at 304.6 °C).

**Figure 6:**
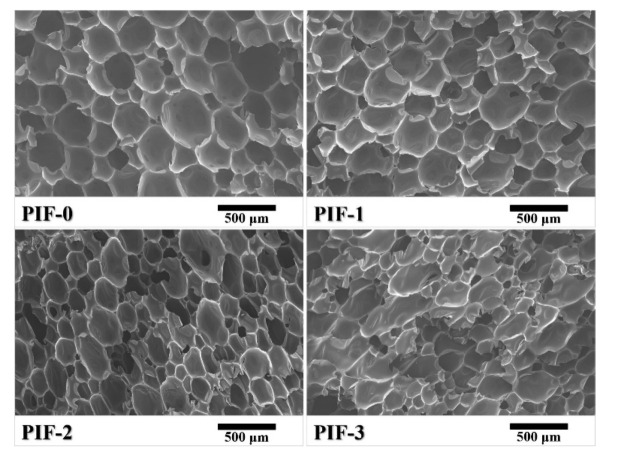
SEM images of the rigid PI foams with different BIA contents.

Closed-cell rigid PI foams—with different molar ratios of BIA/(BIA + PDA) (0% to 30%)—were successfully prepared by thermal foaming of the NPOs. However, when the molar ratio of BIA/(BIA + PDA) exceeded 40%, the NPOs could not be thermally foamed to yield PI foams—attributed to the lack of melt fluidity. Figure 6 compares the micro morphologies of the PI foams. The distribution of the cell sizes of the PI foams ranged from 50 to 600 μm, and most of the cell walls were intact without any broken holes or defects. The cell sizes and uniformity were significantly affected by the BIA content. The average cell diameter decreased with an increase in BIA content, along with the cell size uniformity. Obviously, PIF-3 showed an uneven cell structure.

**Figure 7:**
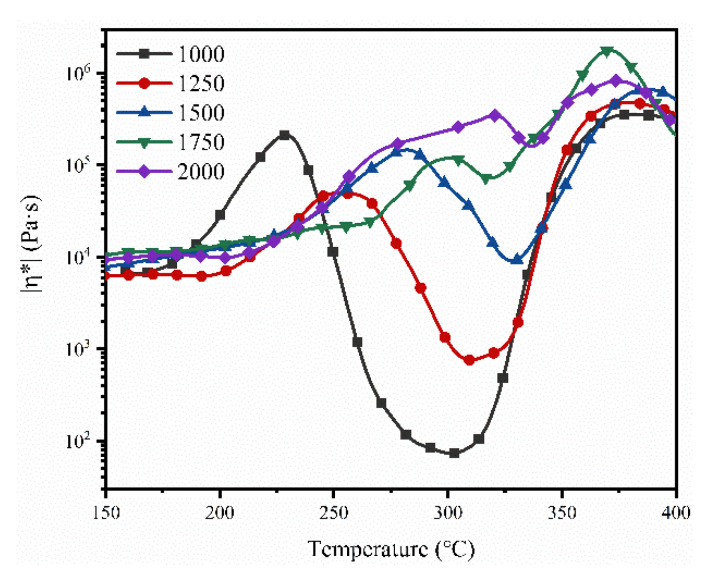
Melt viscosities of NPO-2 with different calcd M_w_.

Figure 7 compares the complex melt viscosity of NPOs with different calcd M_w_. The minimum melt viscosity increased with increasing calcd M_w_. NPO-1000 showed the lowest minimum melt viscosity of 73 Pa·s at 301 °C, compared with NPO-2-1250 (755 Pa·s at 311 °C), NPO-2-1500 (5134 Pa·s at 328 °C), and NPO-2-1750 (72,350 Pa·s at 318 °C). The minimum melt viscosity of NPO-2-2000 was >1.0 × 10^5^ Pa·s at 325 °C, resulting in a poor melt-flowing ability.

**Figure 8:**
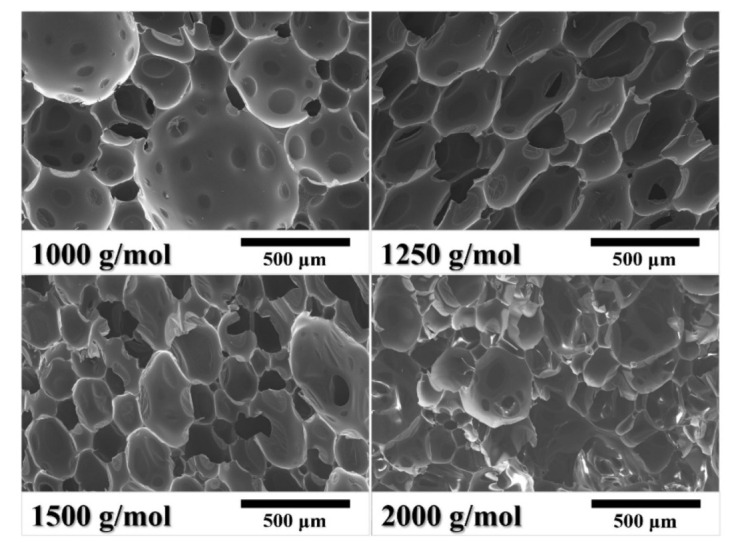
SEM images of rigid PI foams (NPO-2) with different calcd M_w_.

The NPO-2s with a calcd M_w_ of 1000–2000 g/mol could be thermally foamed to produce uniform rigid PI foams. Figure 8 compares the microscopic cell structures of the PI foams. The cell structures and cell diameter of the PI foams clearly changed with increasing calcd M_w_. The cell size of PIF-2-1000 showed an obvious bimodal distribution comprising large-size cells (around 750 μm) and small-size cells (around 200 μm). The melt strength might not be strong enough to maintain cell integrity in the thermal foaming process, resulting in the occurrence of cell merging. PIF-2-1250 and PIF-2-1500 showed relatively uniform cell sizes, probably attributed to the moderate melt strength and blowing agent dosage.

**Figure 9:**
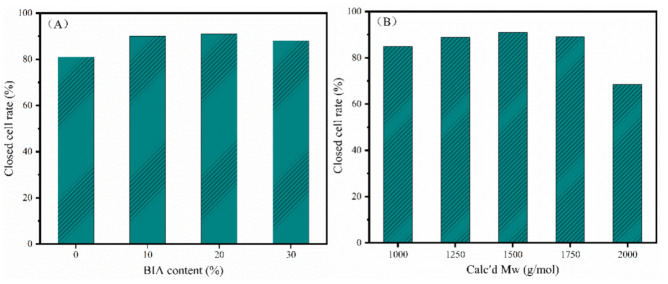
Closed-cell rates of the PI rigid foams according to (**A**) different BIA contents and (**B**) different calcd M_w_.

Closed-cell rigid polymer foams are usually used as a core material of sandwich composites by co-curing of the foam core with carbon fiber prepregs. In this process, the high closed-cell rate is a crucial factor for reducing the weight of the finished product by preventing resin penetration into the foam core. Figure 9A compares the closed-cell rate of the PI foams with different BIA contents. The closed-cell rates of the PI foams were ≥80%, in which PIF-1 and PIF-2 showed closed-cell rates of 90% and 91%, respectively. The rapidly rising viscosity—due to the crosslinking of end-capping groups—might have accounted for the high closed-cell rate. NPO-0, which had the lowest melt viscosity, resulted in a PI foam with the lowest closed-cell rate. However, NPO-3 with the highest melt viscosity also resulted in PIF-3 with a lower closed-cell rate than PIF-1 and PIF-2, indicating that only an appreciable melt viscosity could produce PI foams with a high closed-cell rate. Figure 9B compares the closed-cell rate of the PI foams with different calcd M_w_. The closed-cell rate increased from 85% for PIF-2-1000 to the highest value of 91% for PIF-2-1500. PIF-2-2000 showed a closed-cell rate of <70%. This is consistent with the broken cell structure shown in Figure 8. Overall, the BIA content and the M_w_ had a significant influence on the rheological behavior of the precursor, which in turn affected the foam cell structure. Moderate melt viscosity could contribute to the formation of foam with a uniform cell structure and high closed-cell ratio.

### 3.3. Thermal Properties of the Rigid PI Foams

**Figure 10:**
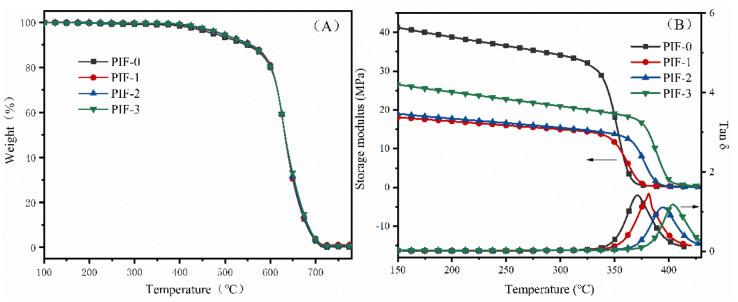
(**A**) TGA and (**B**) DMA curves of the rigid PI foams with different BIA contents.

The thermal properties of the PI foams with different BIA contents are shown in Figure 10, and detailed data are listed in Table 3. No obvious weight loss was detected before 400 °C (Figure 10A), demonstrating the outstanding thermal oxidation stabilities of the foams. The Tg of the PI foams determined by tan δ in DMA was measured at ≥371.4 °C. The PI foams with higher BIA contents exhibited higher Tg values and thermal decomposition temperatures. For instance, PIF-3 showed a Tg of 402.9 °C, much higher than that of PIF-0 (371.4 °C) and PIF-1 (381.8 °C). The Tg of polymers is closely related to the rigidity of the polymer backbone. The BIA monomers used for polymerization enhanced the rigidity of the polyimide backbone. Additionally, the N–H groups in the BIA moiety can form hydrogen bonds with carbonyl groups in the imide ring, restricting the motion of polymer segments and increasing the Tg value [37].

The thermal properties of the PI foams with different calcd M_w_ can be seen in Table 3. Tg values were measured from 387.0 to 409.3 °C, which decreased with an increase in calcd M_w_. For instance, PIF-2-2000 showed a Tg of 387.0 °C, much lower than PIF-2-1000 (409.3 °C) and PIF-2-1500 (395.0 °C), respectively. Thermal decomposition was restricted by increasing the M_w_. The PI foams with lower M_w_ had a higher Tg but a lower thermal stability. Shortening the molecular chain could improve the degree of crosslinking and, thus, contribute to a high Tg. In addition, a lower M_w_ would increase the concentration of end-capping groups in the polymer backbone, resulting in a high ratio of aliphatic structure, which could be easily thermally decomposed at high temperatures [35]. Overall, increasing the BIA content and decreasing the M_w_ of the precursor are two effective methods to enhance the Tg of rigid PI foams.

### 3.4. Mechanical Properties of the Rigid PI Foams

Table 4 summarizes the mechanical properties of the PI foams. PIF-2 possessed the highest strengths, including a compression strength of 1.1 MPa and tensile strength of 0.9 MPa, and the highest elongation at breakage of 6.6%. The elongation at breakage increased from 3.0% for PIF-0 to 5.4% for PIF-1, and to 6.4% for PIF-3 and 6.6% (the highest) for PIF-2, implying that increasing the BIA content enhances the toughness of the foams. PIF-3 showed an obvious deterioration in mechanical properties when the BIA content was increased to 30%, accompanied by an irregular cell structure and lower closed-cell rate.

The PI foam mechanical properties were also influenced by M_w_ (Table 4). All PI foams showed a preferrable compressive strength of ≥1.0 MPa. Increasing the M_w_ can lead to a decrease in tensile strength and enhanced toughness. This might be attributed to the non-uniformity of the cell structure and the decrease in crosslinking density, respectively. Overall, the mechanical properties of rigid PI foams were simultaneously affected by the chemical structure and cell structure of the foams. Generally, toughness can be improved by increasing the BIA content and the M_w_ of the precursors. An intact and uniform cell structure is essential to ensure the preferred mechanical properties of foam materials.

### 3.5. Mechanical Properties of the Closed-Cell Rigid PI Foams with Different Densities

**Figure 11:**
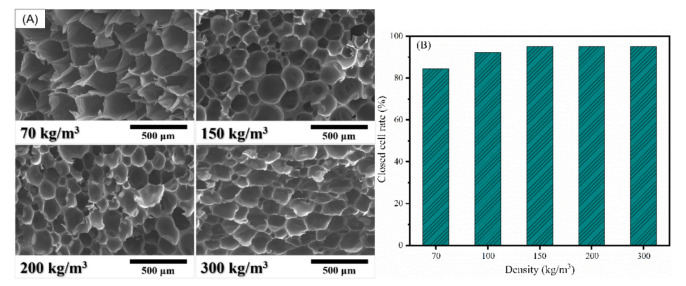
(**A**) SEM images and (**B**) closed-cell rate of the PI foams with different densities.

Density is the most important factor determining the mechanical properties of foam materials [38,39]. A series of PI foams of different densities (70–300 kg/m^3^) were prepared. The precursor selected was NPO-2-1500 with a BIA content of 20% and calcd M_w_ of 1500 g/mol, for which the corresponding PI foam had the best combined properties. Figure 11A compares the SEM images of the PI foams with different densities. It is obvious that all the PI foams showed a uniform cell size distribution, in which the cell size was about 50–300 μm in diameter. In addition, the average cell size decreased with increasing density, and their sizes and their corresponding densities were 223 μm (ρ = 70 kg/m^3^), 216 μm (ρ = 100 kg/m^3^), 145 μm (ρ = 150 kg/m^3^), 136 μm (ρ = 200 kg/m^3^), and 132 μm (ρ = 300 kg/m^3^). The closed-cell rate of the PI foams with different densities can be seen in Figure 11B. All the rigid PI foams exhibited high closed-cell rates of ≥85%, which increased with increasing density. When the density exceeded 100 kg/m^3^, the closed-cell rates were ≥91%.

**Figure 12:**
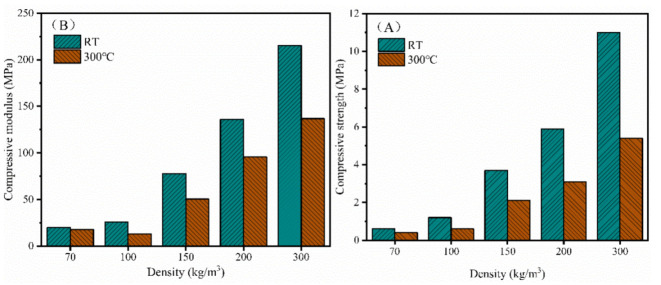
Compressive properties of the PI foams with different densities both at room temperature (RT) and 300 °C: (**A**) compressive strength and (**B**) compressive modulus.

Figure 12 compares the compressive properties of the PI foams with different densities both at room temperature and 300 °C. The compressive strength and modulus increased with the increasing density, which is in accordance with the intensity being related to density as previously reported [38,39]. As the foam density increased from 70 to 300 kg/m^3^, the compressive strength and modulus increased by more than 10 times. The PI foam with 300 kg/m^3^ exhibited excellent mechanical properties with a compressive strength of 11 MPa and a compressive modulus of 215 MPa. Moreover, both the compressive strength and modulus retention at 300 °C were higher than 50%, demonstrating the outstanding thermodynamic properties of the rigid PI foams.

**Figure 13:**
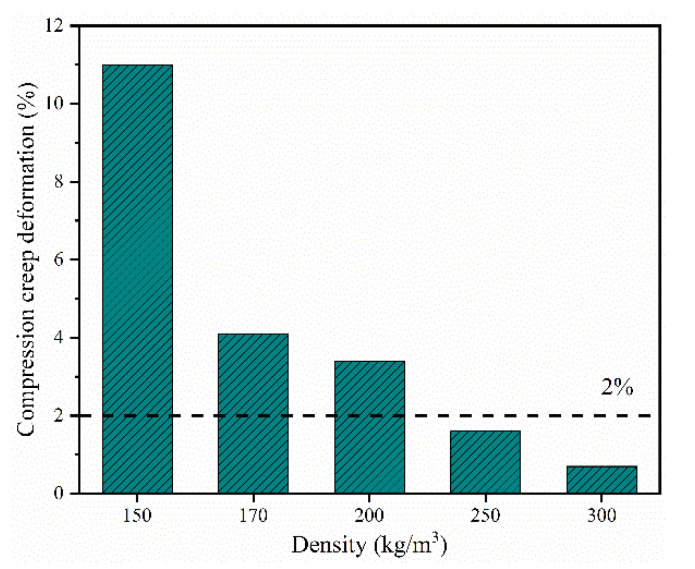
Compression creep deformation of the rigid PI foams with different densities after thermal aging at 320 °C/0.4 MPa for 2 h.

The compression creep properties of the rigid PI foams with different densities are shown in Figure 13. The compression creep deformation decreased sharply with the increasing density. For instance, the creep deformation was 1.6% for a PI foam with 250 kg/m^3^ and 0.7% with a density of 300 kg/m^3^ after thermal aging at 320 °C/0.4 MPa for 2 h, compared with 11% with a density of 70 kg/m^3^. To ensure the stability of the sandwich composite material performance, the compression creep deformation of the foam core material should be lower than 2%. Hence, PI foams with a density of ≥250 kg/m^3^ are suitable as the foam core materials of carbon fiber reinforced polyimide sandwich composites processed at 320 °C.

Table 5 compares the combined properties of PIF-2 in this work with those of other PI foams reported in the literature. PIF-2 shows the most optimally combined properties, including higher Tg values, higher closed-cell rates, and excellent mechanical properties. For instance, PIF-2 has a Tg of 395 °C, 21 °C higher than that of α-BPDA-PDA (374 °C) [31], 31 °C higher than that of α-BPDA-4,4′-ODA (364 °C) [27], 50 °C higher than that of BTDA-4,4′-ODA/BIA (345 °C) [28], and 89 °C higher than that of ODPA-4,4′-ODA/BIA (306 °C) [32], respectively. PIF-2 also has a closed-cell rate of ≥91%, higher than those reported for other PI foams (≤89%). In addition, when the density is similar, the compression performance of PIF-2 is better than for most other PI foams, such as BTDA-MDA/BDM [25], α-BPDA-3,4′-ODA [27], and α-BPDA-4,4′-ODA [27].

## 4. Conclusions

In our study, rigid PI foams with different densities were prepared by thermal foaming of a nadimide end-capped polyimide precursor with an α-BPDA-PDA/BIA structure. The molar ratio of BIA/PDA and the M_w_ of the precursor were found to be two effective factors regulating the performance of PI foams. The prepared PI foams showed excellent combined properties, in which the initial thermal decomposition temperature was ≥490 °C and the Tg was 395.0 °C. PIF-2-1500 (300 kg/m^3^) showed outstanding mechanical properties with a compressive strength of 11 MPa and a compressive modulus of 215 MPa. Moreover, the foams with a density of ≥250 kg/m^3^ were suitable as foam core materials of carbon fiber reinforced polyimide sandwich composites co-cured at 320 °C/0.4 MPa.

## Figures and Tables

**Table 1:** Measured molecular weight of NPO-2 with different calcd M_w_.

Calcd M_w_ (g/mol)	M_n_ (g/mol)	M_w_ (g/mol)	Polydispersity
1000	1644	2385	1.45
1250	1662	2490	1.50
1500	1686	2508	1.49
1750	1688	2528	1.50
2000	1718	2626	1.53

**Table 2:** Minimum melt viscosity of powders NPO with different BIA contents and the corresponding temperature.

NPO	Minimum Melt Viscosity (Pa·s)	Temperature (°C)	n_(BIA)_: n_(BIA+PDA)_
NPO-0	233	317.3	0%
NPO-1	826	324.1	10%
NPO-2	5134	328.9	20%
NPO-3	45,490	304.6	30%

**Table 3:** Thermal properties of polyimide foams with different BIA content and calcd M_w_.

PIFs	TGA		DMA		n_(BIA)_: n_(BIA+PDA)_	Calcd M_w_ (g/mol)
	T_5_ (°C)	T_10_ (°C)	E′ (°C)	tan δ (°C)		
PIF-0	474.1	546.8	339.2	371.4	0%	1500
PIF-1	489.0	557.5	347.2	381.8	10%	1500
PIF-2	492.1	556.9	365.3	395.0	20%	1500
PIF-3	492.1	553.9	376.8	402.9	30%	1500
PIF-2-1000	479.1	521.8	378.2	409.3	20%	1000
PIF-2-1250	488.0	542.5	369.8	397.5	20%	1250
PIF-2-1500	492.1	556.9	365.3	395.0	20%	1500
PIF-2-1750	508.4	574.0	363.5	389.8	20%	1750
PIF-2-2000	512.9	578.5	362.8	387.0	20%	2000

Abbreviations: T_5_, temperature at 5% loss of original weight; T_10_, temperature at 10% loss of original weight; E′, storage modulus; tan δ, tangent of loss angle.

**Table 4:** Mechanical properties of PI foams with different BIA content and different calcd M_w_.

PIFs	Compressive Properties		Tensile Properties		
	Strength (MPa)	Modulus (MPa)	Strength (MPa)	Modulus (MPa)	Elongation at Breakage (%)
PIF-0	1.0	31.0	0.6	21.5	3.0
PIF-1	1.0	35.7	0.6	15.3	5.4
PIF-2	1.1	24.7	0.9	17.0	6.6
PIF-3	0.6	9.8	0.6	11.2	6.4
PIF-2-1000	1.3	27.8	0.6	26.1	2.7
PIF-2-1250	1.1	28.2	1.0	28.6	4.0
PIF-2-1500	1.1	24.7	0.9	17.0	6.6
PIF-2-1750	1.4	35.9	0.6	15.7	4.8
PIF-2-2000	1.0	22.5	0.6	11.9	6.5

**Table 5:** Performance comparison of different PI foams.

PI Foams	Density (kg/m^3^)	Closed-Cell Rate (%)	Tg (°C)	Compressive Strength (MPa)	Compressive Modulus (MPa)
PIF-2 (this work) α-BPDA-PDA/BIA	100	91	395	1.10	25.7
	150	95	395	3.70	77.6
α-BPDA-PDA [27]	100	89	374	1.34	37.1
α-BPDA-3,4′-ODA [27]	100	82	318	0.96	21.1
α-BPDA-4,4′-ODA [27]	100	86	364	0.70	16.3
BTDA-4,4′-ODA [15]	32	32	300	0.30	11.03
ODPA-3,4′-ODA [15]	80	-	237	0.84	6.13
	32	32	237	0.19	3.89
BTDA-MDA/BDM [25]	101	-	285	0.86	10.79
BTDA-4,4′-ODA/BIA [28]	243	-	345	7.45 ^a^	23.8 ^a^
ODPA-4,4′-ODA/BIA [31]	54	-	306	0.71	-
BTDA-4,4′-ODA/DAPBO [32]	77	-	368	1.03	-

Compressive properties were tested at 10% strain. ^a^ These compressive properties were measured at 15% strain. Abbreviations: ODPA, 4,4′-oxydiphthalic anhydride; 3,4′-ODA, 3,4′-oxydianiline; 4,4′-ODA, 4,4′-oxydianiline; BTDA, 3,3′,4,4′-benzophenonetetracarboxylic dianhydride; MDA, 4,4-diaminodiphenyl methane; BDM, 4,4′-bismaleimidediphenylmethane; PDA, p-phenylenediamine; α-BPDA, 2,3,3′,4′-biphenyl tetracarboxylic dianhydride; BIA, 2-(4-aminophenyl)-5-aminobenzimidazole; m-PDA, m-phenylenediamine; DAPBO, 2-(4-aminophenyl)-5-aminobenzoxazole.

## Data Availability

The data presented in this study are available on request from the corresponding author.
